# Investigation of the Adsorption Process of Chromium (VI) Ions from Petrochemical Wastewater Using Nanomagnetic Carbon Materials

**DOI:** 10.3390/nano12213815

**Published:** 2022-10-28

**Authors:** Wei Long, Zhilong Chen, Xiwen Chen, Zhanye Zhong

**Affiliations:** 1 Guangdong Provincial Key Laboratory of Petrochemical Pollution Process and Control, Guangdong University of Petrochemical Technology, Maoming 525000, China; 2 College of Chemistry, Guangdong University of Petrochemical Technology, Maoming 525000, China

**Keywords:** adsorption, petrochemical wastewater, nano magnetic carbon materials, Cr(VI) ions

## Abstract

Magnetic mesoporous carbon (MMC) and magnetic activated carbon (MAC) are good functionalized carbon materials to use when applying environmental techniques. In this work, a series of efficient magnetic composite adsorbents containing Fe_3_O_4_ and carbon were prepared successfully and used for the adsorption of Cr(VI) ions in petrochemical wastewater. The morphology and structure of these magnetic adsorbents were characterized with FTIR, TG, XRD, VSM, BET, and SEM technologies. The effect of different factors, such as pH, adsorption time, initial Cr(VI) ions’ concentration, Fe_3_O_4_ loading, and adsorption time, on the adsorption behavior were discussed. The results showed that the 8%Fe_3_O_4_@MMC adsorbent exhibited a high removal rate, reutilization, and large adsorption capacity. The corresponding adsorption capacity and removal rate could reach 132.80 mg·g^−1^ and 99.60% when the pH value, adsorption time, and initial Cr(VI) ions’ concentration were 2, 180 min, and 80 mg·L^−1^ at 298 K. Four kinds of adsorption isotherm models were used for fitting the experimental data by the 8%Fe_3_O_4_@MMC adsorbent at different temperatures in detail, and a kinetic model and thermodynamic analysis also were performed carefully. The reutilization performance was investigated, and the Fe_3_O_4_@MMC adsorbent exhibited greater advantage in the adsorption of Cr(VI) ions. These good performances can be attributed to a unique uniform pore structure, different crystalline phases of Fe_3_O_4_ particles, and adsorption potential rule. Hence, the 8%Fe_3_O_4_@MMC adsorbent can be used in industrial petrochemical wastewater treatment.

## 1. Introduction

Nowadays, environmental pollutants are concerning to more and more scientists, as they threaten human survival on the earth [1,2]. Heavy-metals pollution is especially a large challenge to human and animal health, including cadmium, lead, chromium, mercury, zinc, and copper ions, as all of them can easily accumulate in the body [3]. The degradation process of metal ions is very difficult, so the heavy-metals pollution also has attracted the attention of many experts [4,5,6].

Petrochemical wastewater is a kind of typical pollutant, including many heavy-metal ions and other toxic organic substances. For the continuous petroleum refining, petrochemical wastewater has to be discharged continuously [7]. Although the wastewater-treatment technology and devices have been enforced by the government, there is still a certain concentration of heavy metals discharged into the environment [8]. Owing to the development of industry, excessive use of landfills, and stacking of civil solid wastes, large amounts of heavy metals also enter rivers or lakes [9]. Hence, the recovery of heavy metals from wastewater has become a major topic of research in water treatment.

Chemical precipitation, membrane filtration, and ion exchange are commonly used methods for wastewater treatment, but adsorption has been shown to be an economical alternative for removing trace metals from wastewater [10,11]. Activated carbon is a commonly good adsorbent for many materials, such as as gas, dye, metal ions, and organic substances; it is used for water treatment widely due to the stronger physical adsorption [12,13,14,15]. However, the asymmetrical pore structures of activated carbon have no advantage for selectivity adsorption and rapid desorption [16]. Mesoporous carbon materials are popular in adsorption and catalysis industries due to well-defined structural, textural, and morphological properties [17,18]. In many applications, chemical modification of the mesoporous carbon material surfaces can be used to provide a desired functionality, surface reactivity, or create active sites for the grafting of desirable compounds or coating special particles [19,20].

A number of different typical carbon materials are reported for the good adsorption of Cr(VI) ions in Table 1 [21,22,23,24,25,26,27,28,29]. The adsorption capacity for Cr(VI) ions are not good, and the acidic solution is more suitable compared to alkalic solution. Xu et al. [30] found the adsorption capability reached 165.93 mg·g^−1^, and the adsorption rate reached 0.0029 g·mg^−1^·min^−1^ by mPC adsorbent. Mesoporous carbon nitride (MCN) showed higher adsorption capability than activated carbon (AC) at pH 5.7 [31]. Ordered mesoporous carbon (OMC) can exhibit a great adsorption capacity for As(V) and Cr(VI) ions simultaneously, and this is ascribed to the high electron transfer rate and low electrical resistance at a pH of 3 [32]. The Fe_3_O_4_ nanoscale particles on the surface of carbon microspheres can form outer-sphere complexes with Cr(VI) and reduce Cr(VI) to Cr(III) [33], which is beneficial to remove metal ions. More and more research shows that functionalized magnetic nanocarbon material exhibited great advantage to adsorb heavy-metal ions [34,35,36]. Although carbon materials can be used for the removal of pollutants such as cytochrome C [37], carbon dioxide [38], phenol [39], and reactive dyes [40], the adsorption is not uniform, and the preparation processes of many adsorbents are complicated and expensive. Hence, the development of cheap and simple magnetic carbon adsorbent still is of great significance.

Thus, this study aimed to investigate two kinds of magnetic composite carbon materials, modified mesoporous carbon supported Fe_3_O_4_ and modified activated carbon supported Fe_3_O_4_, which all can be used to evaluate the adsorbent potential of Cr(VI) from the real petrochemical wastewater. Compared to modified-activated-carbon-supported Fe_3_O_4_, the Fe_3_O_4_@MMC adsorbent can perform better adsorption performance. The optimum adsorption conditions and adsorption equilibrium were obtained, and the adsorption behavior features were analyzed by using isothermal models. The characterization, kinetics, and thermodynamics were discussed carefully.

## 2. Materials and Methods

### 2.1. Chemicals and Instruments

Activated carbon and mesoporous carbon powder were purchased from Suzhou Tan-Feng science and technology Co., Ltd. (Suzhou, China), and further purification or modification were performed in the laboratory. Real petrochemical wastewater samples were collected from the discharged sewage of Maoming petrochemical refining Co., Ltd. (Maoming, China).

The other chemicals in this study were consumed as provided by suppliers, without any further modifications. (NH_4_)_2_FeSO_4_·6H_2_O, polyethylene glycol, K_2_Cr_2_O_7_, FeCl_3_·6H_2_O, 1,5-diphenyl carbazide, and FeCl_2_·4H_2_O were purchased from Shanghai McLean Biochemical Technology Co., Ltd. (Shanghai, China). Hydrochloric acid, acetic acid, sulfuric acid, ethanol, sodium hydroxide, and ammonia were provided by Tianjin Damao Chemical Reagent Co., Ltd. (Tianjin, China).

### 2.2. Preparation of Adsorbent

Modification of carbon material was performed as follows. Firstly, 1.0 g activated carbon or mesoporous carbon powder was calcined for 3 h, at 600 °C, under the protection of N_2_ gas in order to remove the organic impurities. Then, the powder was washed by distilled water five times and dried in air for 8 h at 110 °C in order to remove the inorganic impurities. Then 1.0 g of carbon material was transferred into the flask, and 50 mL of acetic acid solution (85 wt.%) was added into the flask and stirred for 2 h at room temperature. The mixture solution was further refluxed for 4 h, at 85 °C, with continuous stirring, so more defects and carboxyl groups appeared on the surface of the carbon material. After being cooled to the room temperature, the black powder was separated from the mixture solution, and it was washed with distilled water till the pH was 7.0. The black powder was vacuum dried at 60 °C for 8 h, and it was labeled as MAC (modified activate carbon) or MMC (modified mesoporous carbon).

Magnetic adsorbent was prepared as follows: 5.00 g FeCl_3_·6H_2_O, 3.68 g FeCl_2_·4H_2_O, and 25 mL H_2_O were mixed into a flask and stirred for 30 min. Then a certain mass of MMC or MAC powder and 5 mL polyethylene glycol solution (5 wt.%) were added into the mixture solution and stirred for 2 h at 60 °C. Next, some sodium hydroxide solution (1 mol·L^−1^) was dropped into the mixture solution slowly, till the pH value reached 11, with continuous stirring, at 60 °C. After the black solution was cooled to the room temperature, the mixture was aged for 2 h in a microwave washer and dried in air overnight at 90 °C and crushed. Then it was washed with distilled water and dried in air for 8 h at 90 °C. It was labeled as x%Fe_3_O_4_@MAC or x%Fe_3_O_4_@MMC adsorbent; x% is the mass percentage of Fe_3_O_4_ in the magnetic adsorbent.

### 2.3. Characterization

The X-ray diffraction (XRD) pattern of the sample was recorded by the Bruker D8 Advance device (Bruker Corporation, Berlin, Germany) with Cu-Kα radiation (λ = 1.54 A) between 20 and 70° (2θ) at 40 kV and 40 mA. FTIR spectra were recorded as the KBr films in the range of 500–4000 cm^−1^ on a PerkinElmer spectrum One (B) spectrometer (PerkinElmer, Tokyo, Japan). The BET characterizations of the samples were obtained by the nitrogen adsorption–desorption on a Quantachrome NOVA-2200E automated gas sorption system (Quantachrome Instruments, Washington, DC, USA), and the specific surface areas and average pore size were calculated. The thermal stability of the samples was analyzed by Netzsch 209C thermogravimetric (TG), (Netzsch, Beijing, China)., with a temperature range of 303–1173 K and a heating rate of 10 K·min^−1^ under nitrogen atmosphere. A vibrating-sample magnetometer (VSM) (EG & G Princeton Applied Research Vibrating Sample Magnetometer, Model 155, Washington, DC, USA) was used at room temperature to characterize the magnetic properties of magnetic material microspheres. The morphology of the samples was observed by scanning electron microscope (SEM, JEOL 6500F, JEOL, Berlin, Germany). The analysis of the amount of Fe of the adsorbent was determined by inductively coupled plasma–atomic emission spectrometry (IRIS Intrepid II XSP ICP–OES, Washington, DC, USA).

### 2.4. Adsorption and Desorption Study

Firstly, K_2_Cr_2_O_7_ standard solution was used to evaluate the accuracy of the detection testing method. Many approximate conditions, such as the pH, adsorption time, and adsorbent dosage, were obtained in K_2_Cr_2_O_7_ standard solution. Then the adsorption tests were conducted by using aqueous solutions of the real petrochemical wastewater, and the adsorption conditions were referred in K_2_Cr_2_O_7_ standard solution.

For adsorption study, 0.03 g adsorbent was dispersed into 50 mL real petrochemical wastewater containing chromium(VI) ions. After the value of pH was adjusted to 2.0, the zeta potential of the solution was detected carefully. Zero charge of solution was adjusted carefully before every adsorption experiment. Then the adsorption was performed in a continuously vibrating shaker, under 298 ~ 318 K (the shake speed was fixed at 400 rpm). The solution was filtered after a certain adsorption time, and the liquid sample was sent to be analyzed.

The adsorbate Cr_2_O_7_^2−^ was estimated by using the Chinese National Standards GB/T 7467-87 method [41] (λ = 540 nm,1,5-diphenyl carbazide is chromogenic agent). The pH was adjusted by using sulfuric acid or ammonia, and all pH measurements were carried out by using a digital pH meter. The mixture was filtered with filter paper, and the residual Cr(VI) concentration in the filtrate was determined by a microspectrophotometer device, using a matched quartz cells. In order to evaluate the accuracy, some samples were obtained by atomic adsorption (GBS, SENS AE), according to method 3110 of APHA [42].

The x%Fe_3_O_4_@MMC or x%Fe_3_O_4_@MAC is used for chromium(VI) ions adsorption, and the influencing factors, including the initial chromium(VI) ions’ concentration, adsorption time, Fe_3_O_4_ loading_,_ the pH value, and temperature on the adsorption behavior of the Fe_3_O_4_@MMC or Fe_3_O_4_@MAC adsorbents for chromium(VI) ions, were investigated. At the same time, the adsorption amount and removal rate are determined by the following equations:

(1) $$Q_{e}=\frac{(C_{0}-C_{e})\times V}{m}$$

(2) $$R=\frac{\left(C_{0}-C_{e}\right)}{C_{0}}\times 100\%$$

where Q_e_ (mg·g^−1^) is the adsorption amount of the adsorbent for chromium(VI) in the solution; C_0_ is the concentration of chromium(VI) ions (mg·L^−1^) in the solution before adsorption; C_e_ is the equilibrium concentration of chromium(VI) ions (mg·L^−1^) after adsorption; m and V are the substrate mass (g) of the adsorbent and solution volume (L); and R represents the removal rate (%) of chromium(VI) in the solution.

Additional, for the desorption study, 0.03 g of Fe_3_O_4_@MMC or Fe_3_O_4_@MAC was dispersed into 50 mL of petrochemical wastewater solution containing chromium(VI) ions 80 mg·L^−1^ at 298 K, pH 2, and stirred for 180 min; the saturated Cr(VI)-adsorbed Fe_3_O_4_@MMC or Cr(VI)-adsorbed Fe_3_O_4_@MAC was separated from the solution. The Cr(VI) ions were desorbed from the adsorbent with 1 mol·L^−1^ H_2_SO_4_ as an eluent. Then the desorbed Fe_3_O_4_@MMC was washed with distilled water, dried, and reused under the same conditions to study the reutilization performance of the Fe_3_O_4_@MMC or Fe_3_O_4_@MAC adsorbent.

### 2.5. Data Processing

The biosorption capacity (Q_e_) was determined according to Equation (1), and the results obtained were plotted as a function of the equilibrium concentration in the liquid phase (C_e_). The equilibrium data were analyzed according to the Langmuir, Freundlich, Temkin, and Dubinin–Radushkevich isotherm models. All the models presented in Table 2 were fitted to the experimental data by the nonlinear regression method of the software Statistica 7.5 (Statisticsoft, Tulsa, OK, USA), using the Levenberg–Marquardt algorithm [43].

The adsorption tests were carried out with 50 mL of solution with pH corrected for the same conditions as the isotherms, with different dosages of adsorbent (0.02 to 0.2 g) being added and kept under agitation at 400 rpm.

Adsorption kinetics models were investigated by the pseudo-first-order and the pseudo-second-order model; the corresponding equations are as follows.

(3) $$Q_{t}=Q_{e}(1-e^{-k_{1}t})$$

(4) $$Q_{t}=\frac{Q_{e}^{2}k_{2}t}{1+Q_{e}k_{2}t}$$

where Q_t_ represents the adsorption capacity of adsorbent at time, t, and the unit is mg·g^−1^; Q_e_ represents the adsorption capacity of adsorbent at equilibrium time, and the unit is mg·g^−1^; t represents the adsorption time, the unit is min; and k_1_ and k_2_ represent the pseudo-first-order model rate constant according to Equation (3) and the pseudo-second-order model rate constant according to Equation (4), respectively, and the unit is g·mg^−1^·min^−1^.

The thermodynamic analysis was investigated by the following equations:

(5) $$\ln(\frac{Q_{e}}{C_{e}})=-\frac{\Delta H^{0}}{RT}+\frac{\Delta S^{0}}{R}$$

(6) $$\Delta G=-RT\cdot \ln(\frac{Q_{e}}{C_{e}})$$

Many experimental values (Q_e_ and C_e_) are detected at different temperatures. Then the linear fitting and analysis were performed for the calculation of thermodynamics parameters (ΔH^0^, ΔS^0^, and ΔG). ΔH^0^ represents the enthalpy change according to Equation (5), ΔS^0^ represents the entropy change according to Equation (5), and ΔG represents the Gibbs free energy change, according to Equation (6).

## 3. Results and Discussion

### 3.1. Characterization of Adsorbents

**Figure 1:**
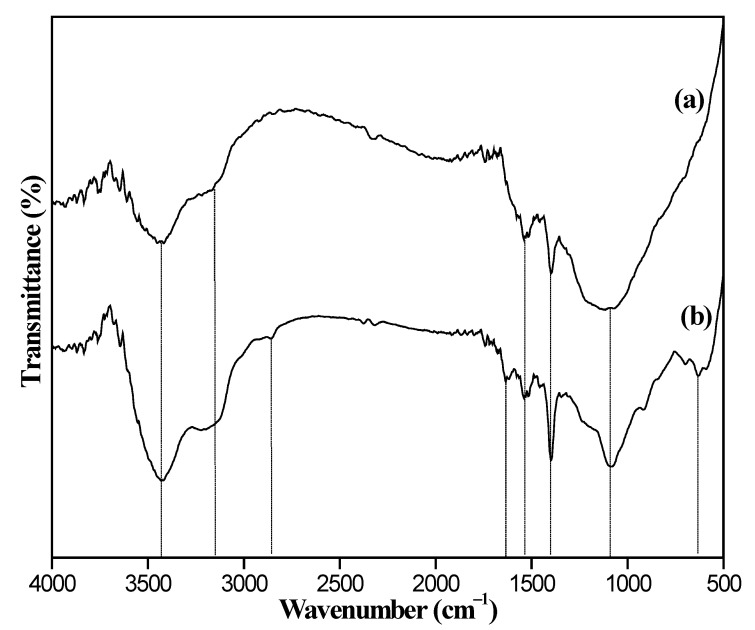
FTIR spectrum of adsorbent samples: (**a**) MMC and (**b**) 8%Fe_3_O_4_@MMC.

**Figure 2:**
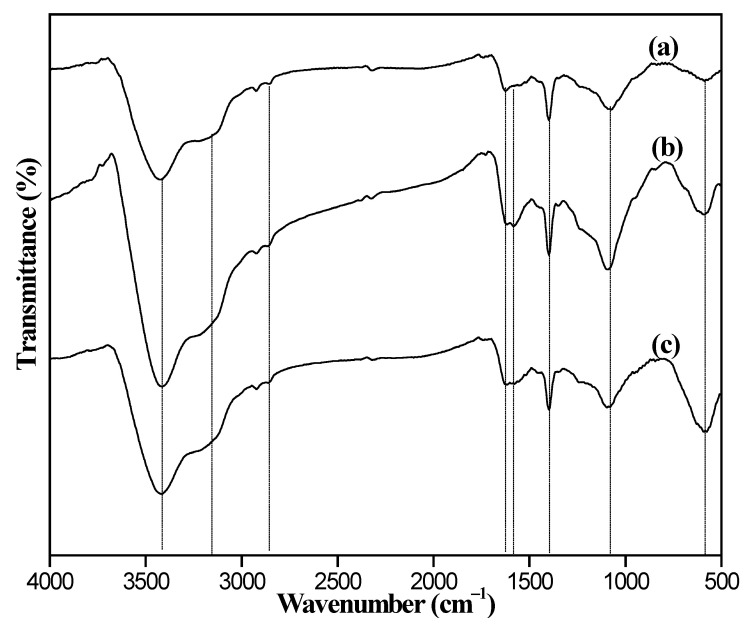
FTIR spectrum of adsorbent samples: (**a**) 6%Fe_3_O_4_@MAC, (**b**) 8%Fe_3_O_4_@MAC, and (**c**) 10%Fe_3_O_4_@MAC.

The FTIR spectras of MMC and 8%Fe_3_O_4_@MMC samples are shown in Figure 1, and there are some characteristic adsorption peaks in the curves. The obvious peak at 3427.8 cm^−1^ is ascribed to asymmetric stretching vibration of the -OH bond in the crystal water molecule. The weak peak at 3152.5 cm^−1^ is ascribed to the asymmetric stretching vibration of the -OH bond in the carboxyl group on the surface of the adsorbent. The weak peak at 2847.7 cm^−1^ is ascribed to the stretching vibration of the -C-H bond, but the similar signal of this peak is inapparent in the curve of MMC. The small peak at 1644.3 cm^−1^ belongs to the asymmetry stretching vibration of the -C=O bond and it is so weak in the curve of MMC due to the acetic acid modified extent is not deep. The small peak at 1531.8 cm^−1^ belonged to the bending stretching vibration of the C-O-C bond, and the distinct peak at 1396.9 cm^−1^ is the characteristic peak of -COOH on the surface of adsorbent. The broad peak at 1071.4 cm^−1^ is ascribed to the stretching vibration of C-H in -CH_3_, -CH_2_, or -CH. The acute adsorption peaks at 593.6 cm^−1^ belonged to the stretching vibration of the Fe-O bond of Fe_3_O_4_ in the adsorbent, which is in accordance with the literature result [44]. The FTIR spectrum of adsorbent samples, including 6%Fe_3_O_4_@MAC, 8%Fe_3_O_4_@MAC, and 10%Fe_3_O_4_@MAC, are displayed in Figure 2; the strength of the stretching vibration of Fe-O bond at 593.6 cm^−1^ was enhanced with the increasing increments of Fe_3_O_4_. Hence, there is a certain amount of -COOH on the surface of the adsorbent, and Fe_3_O_4_ particles are loaded successfully.

**Figure 3:**
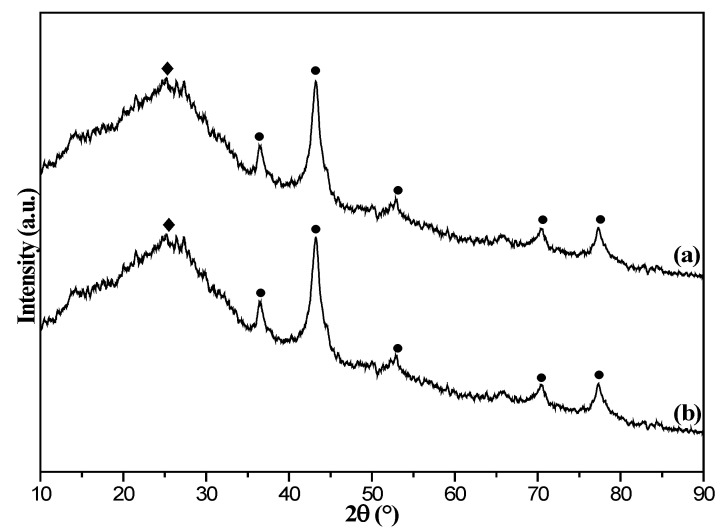
X-ray diffraction (XRD) patterns of 8%Fe_3_O_4_@MMC adsorbent: (**a**) fresh and (**b**) after adsorption.

The XRD spectra of the samples (8%Fe3O4@MAC, fresh and after adsorption) are shown in Figure 3. The characteristic diffraction peaks at 2θ = 30.36°, 35.76°, 43.47°, 53.94°, 57.51°, and 63.17° are ascribed to [220], [311], [400], [422], [440] and [511] crystalline phases of Fe_3_O_4_ species [45]. It showed that the Fe_3_O_4_ cubic crystal characteristics of particles did no change after adsorption. The sharp diffraction peak at 2θ =35.76° indicated that the [311] crystalline phase was the major component. The average particle size of Fe_3_O_4_ species was nearly 24.8 nm, which was calculated from the XRD data based on the Scherrer equation [46].

**Figure 4:**
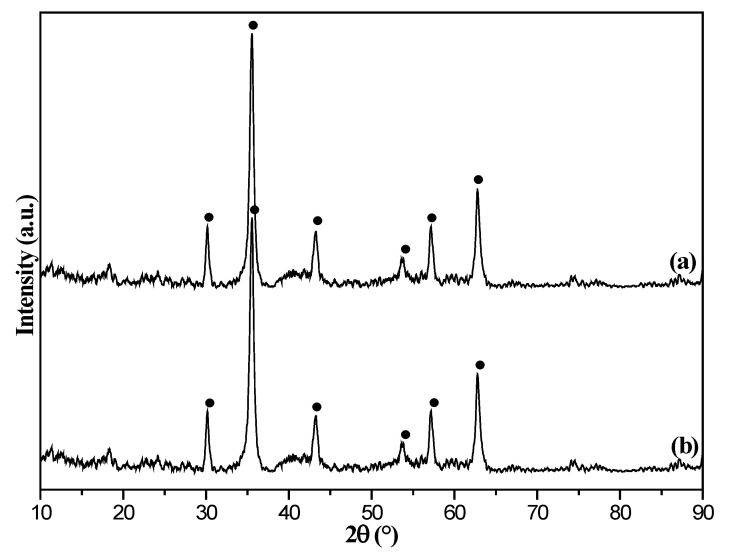
X-ray diffraction (XRD) patterns of 8%Fe_3_O_4_@MAC adsorbent: (**a**) fresh and (**b**) after adsorption.

The XRD spectra of the samples (8%Fe_3_O_4_@MMC, fresh and after adsorption) are shown in Figure 4. The characteristic diffraction peaks at 2θ = 37.08°, 43.45°, 53.91°, 71.52°, and 78.97° correspond to [311], [400], [422], [513], and [621] crystalline phases of Fe_3_O_4_ species. The obvious characteristic diffraction peaks at 2θ = 26.6° was ascribed to the mesoporous carbon [47], and weak peak at 2θ = 30.36° was covered. It showed that the Fe_3_O_4_ cubic crystal characteristics of the particles did not change after adsorption. A sharp diffraction peak at 2θ =35.76° indicated that [400] crystalline phase was the major component. The average particle size of Fe_3_O_4_ species was nearly 18.6 nm, which was calculated from the XRD data based on the Scherrer equation [46]. Hence, the average particle size of Fe_3_O_4_ species was smaller, and magnetic particles almost are not lost after adsorption in the 8%Fe_3_O_4_@MMC adsorbent.

The textural properties, such as the BET surface area, average pore size, pore volume, and average particle size, of the samples are shown in Table 3. It was clear that the surface area and pore volume of MAC and Fe_3_O_4_@MAC samples have an obvious advantage, but the average pore size was low, and the average particle size was large. MMC and Fe_3_O_4_@MMC samples showed a different situation, the average pore size was larger, and the average particle size was smaller. The pore volume enlarged slightly with the addition of the appropriate amount of Fe_3_O_4_ particles, indicating that the magnetism particles preferentially adhered to the inside of the pore channels. Too many magnetism particles might have blocked some pore channels, so the pore volume became smaller in the 15%Fe_3_O_4_@MAC or 15%Fe_3_O_4_@MMC adsorbent. As the increments of the Fe_3_O_4_ mass ratio in the adsorbent increased, the average pore size cut down obviously, and this was ascribed to the fact that the Fe_3_O_4_ particles might block some pore channels. We noted that the surface area of the 8%Fe_3_O_4_@MMC absorbent was as high as 452.3 m^2^·g^−1^, which was the maximum in magnetic mesoporous adsorbent materials. The pore volume was larger than the other magnetic mesoporous adsorbent materials, and the average pore size was more than magnetic activated adsorbent materials; this was possibly a great advantage.

N_2_ adsorption–desorption isotherms and pore size distributions of 8%Fe_3_O_4_@MMC adsorbent are displayed in Supplementary Figures S1 and S2. The hysteresis loop is type H4 according to the IUPAC classification; it means that the adsorbent has the feature of highly ordered pores. The pore diameter distribution is centered at 6.8 nm, so the mesoporous structure of adsorbent was affirmed, and Fe_3_O_4_ loading had not changed. The N_2_ adsorption–desorption isotherms and pore size distributions of 8%Fe_3_O_4_@MAC adsorbent are displayed in Supplementary Figures S3 and S4. The hysteresis loop is type H1 according to the IUPAC classification, and lots of micropores and homogeneous pore distributions (including different pore sizes) were confused. However, it was consistent with the results from the literature [48,49].

**Figure 5:**
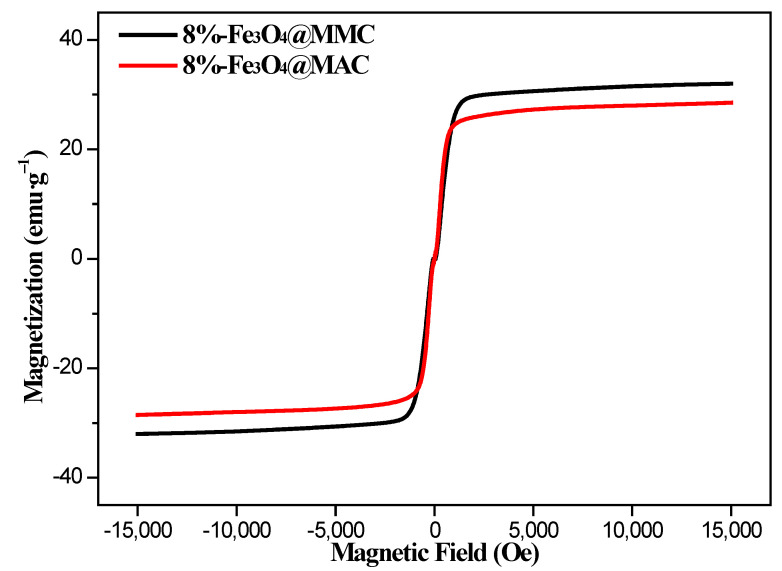
VSM magnetization curves of samples.

Figure 5 exhibits the VSM magnetization curves of the samples that were tested at room temperature. Both the 8%Fe_3_O_4_@MAC and 8%Fe_3_O_4_@MMC adsorbent showed weak magnetism. The magnetization saturation (Ms) values of 8%Fe_3_O_4_@MAC were lower than 8%Fe_3_O_4_@MMC, as seen from Figure 5, and highly ordered mesoporous pores were beneficial for the loading of nano magnetic particles.

**Figure 6:**
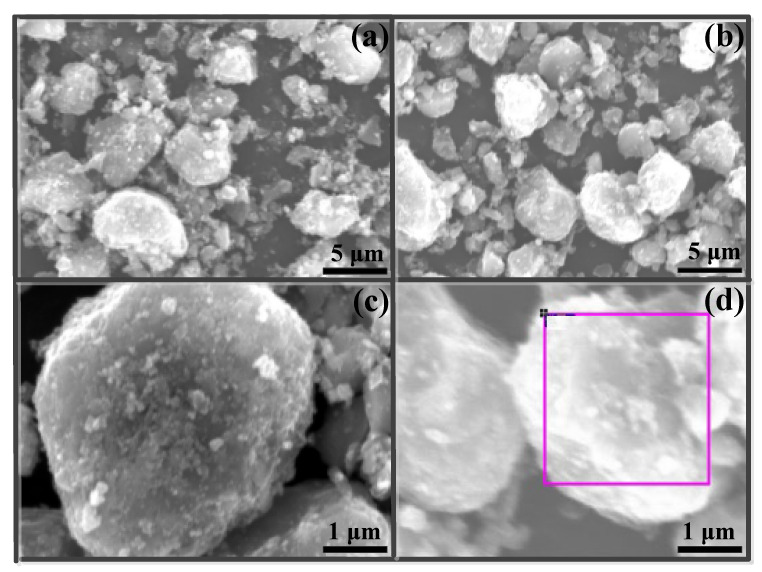
SEM images of 8%Fe_3_O_4_@MMC adsorbent: (**a**) fresh, ×5000; (**b**) after adsorption, ×5000; (**c**) fresh, ×25,000; and (**d**) before adsorption, ×25,000, pink frame represents selected area for EDS.

The SEM images of 8%Fe_3_O_4_@MMC before and after adsorption are displayed in Figure 6. The irregular block-structure characteristics of the fresh absorbent are shown in Figure 6a; the surface is smooth, and some Fe_3_O_4_ particles were loaded. After adsorption, the particles became larger in size, and the surface became brighter, which is displayed in Figure 6b clearly. The enlarged picture of the surface of a single particle is shown in Figure 6c; the surface of the particle is dull and coarse. However, this is different for the adsorbent after adsorption at the same magnification, as is display in Figure 6d. The surface of the particles became brighter, and an area was selected for surface-element EDS analysis; the results are listed in Table 4.

We found that the atom percentages of Fe and O are 3.12% and 43.69%, respectively (in Table 4). The ratio of Fe/O is not as 3:4 due to many -COOH groups existing on the surface of the adsorbent. The weight percentage and atom percentage of C and O are great from the SEM–EDS analysis results in Table 4, as is consistent with the prepare progress of the adsorbent. There was trace Cr percentage in the result due to the strong adsorption behavior in the petrochemical wastewater. Hence, the brighter surface in the SEM image (Figure 6d) was attributed to the strong adsorption behavior between active sites and Cr(VI) ions in solution.

**Figure 7:**
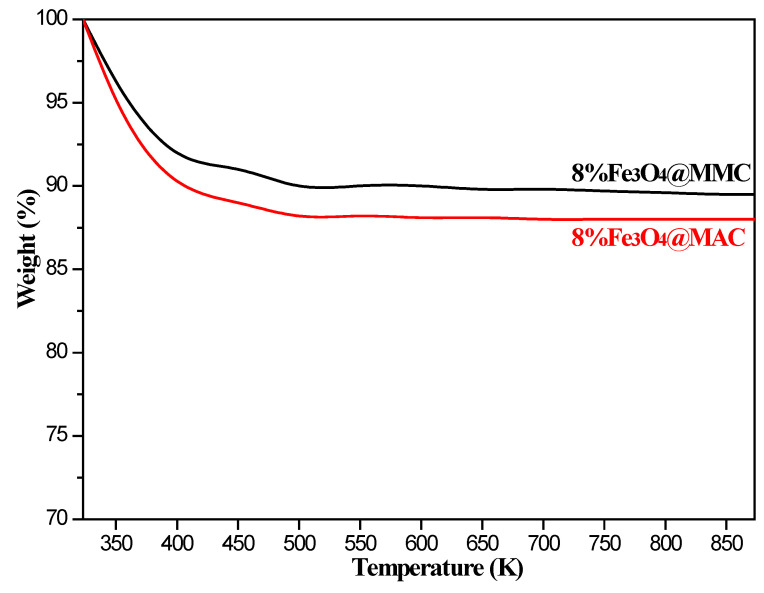
TG curves of samples.

The TG curves of samples are also displayed in Figure 7; the thermostability of 8%Fe_3_O_4_@MMC is better than 8%Fe_3_O_4_@MAC. The slight weight loss is mainly attributed to the loss of water and carboxyl on the surface of the adsorbent. The weight loss rate is less than 10%, which is in accordance with the characteristics of inorganic adsorption materials.

### 3.2. Effect of pH Value and Initial Cr(VI) Ions’ Concentration

**Figure 8:**
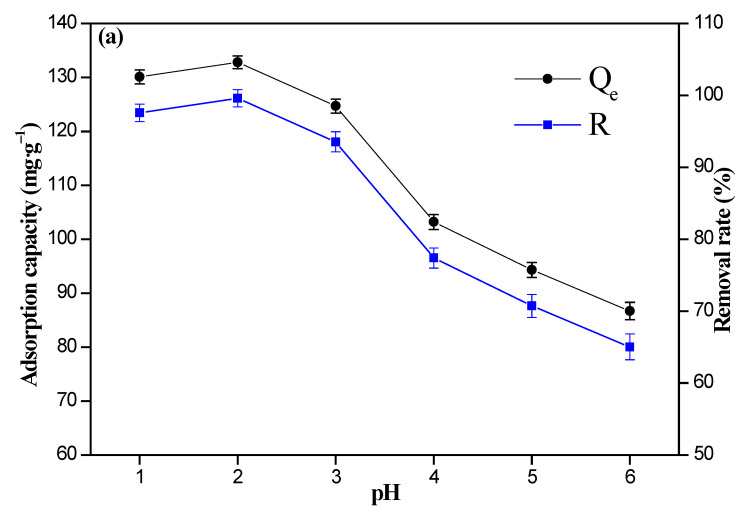

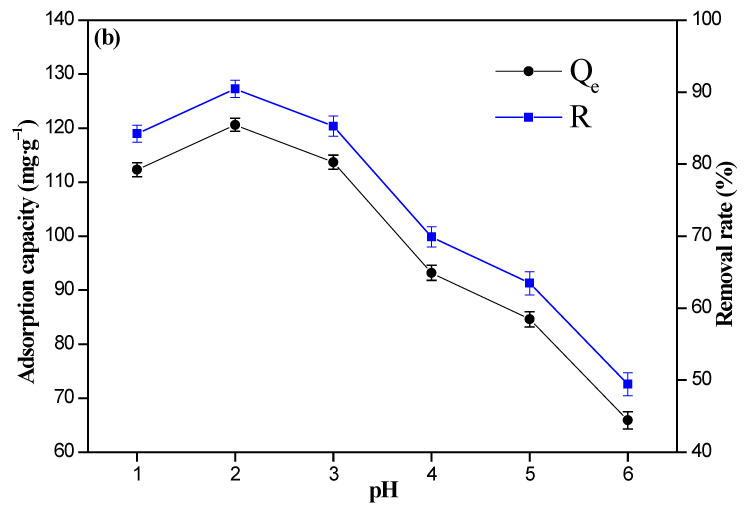
Effect of pH value on the sorption of Cr(VI) ions: (**a**) 8%Fe_3_O_4_@MMC and (**b**) 8%Fe_3_O_4_@MAC. Adsorption conditions: the initial Cr(VI) ions’ concentration, 80 mg·L^−1^; volume, 50 mL; adsorbent dosage, 0.03 g; temperature, 298 K; adsorption time, 180 min.

The pH and the initial Cr(VI) ions’ concentration are very important parameters for the adsorption behavior, and the adsorption capacities of the 8%Fe_3_O_4_@MMC and 8%Fe_3_O_4_@MAC adsorbent are described in Figure 8a,b, respectively.

The adsorption capacity of 8%Fe_3_O_4_@MMC and 8%Fe_3_O_4_@MAC showed that a similar variation tendency, adsorption capacity of the adsorbent, and removal rate reached the maximum when the pH value was 2.0. Considering that the occurrence of the hydrolysis reaction of Cr(VI) ions was easy, it was not essential for the alkaline solution was to be attempted. The following transition of Cr(VI) ions is inevitable, and this transition was related to the acidity of the solution [50]:

Cr_2_O_7_^2−^ + H_2_O ⇆ 2 HCrO_4_^−^ K_a_ = 10^−2.2^

HCrO_4_^−^ ⇆ CrO_4_^2−^ + H^+^ K_a_ = 10^−6.49^

In the low concentration of H^+^ solution, the major Cr_2_O_7_^2−^ ions were translated into CrO_4_^2−^ ions, so the adsorption behavior by this adsorbent varied, and the adsorption capacity reduced. In the high-concentration H^+^ solution, too many hydrogen ions may have occupied adsorption-active sites, so the intense competition between Cr(VI) ions and H^+^ was unfavorable to adsorption. Hence, the optimal pH value of solution was 2.0, and this parameter was fixed for further investigation.

**Figure 9:**
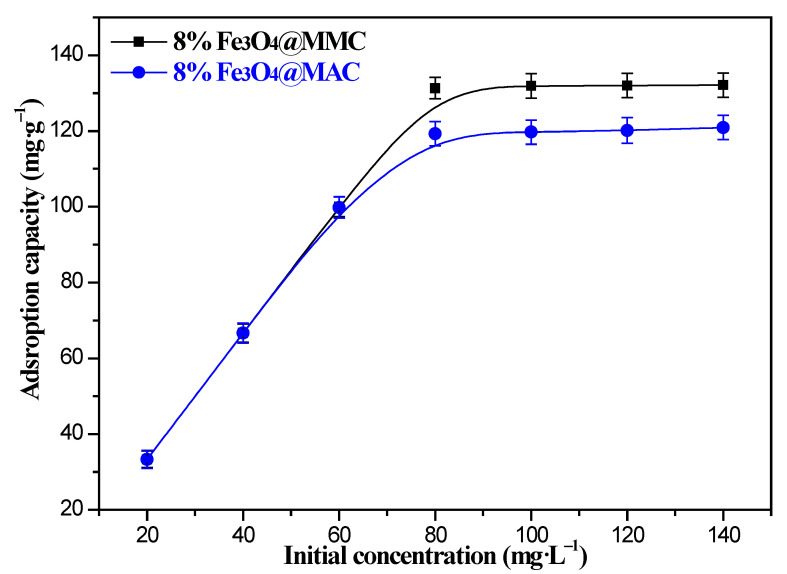
Effect of initial Cr(VI) ions’ concentration on the sorption. Adsorption conditions: volume, 50 mL; pH, 2.0; adsorbent dosage, 0.03 g; temperature, 298 K; adsorption time, 180 min.

The effects of the initial Cr(VI) ions’ concentration on the adsorption are displayed in Figure 9. We found a clear upward trend with the increment of initial Cr(VI) ions’ concentration.

The adsorption capacity of the 8%Fe_3_O_4_@MMC and 8%Fe_3_O_4_@MAC adsorbents was almost unchanged since the initial Cr(VI) ions’ concentration was more than 80 mg·L^−1^, and it is very clear that the adsorption capacity of the 8%Fe_3_O_4_@MMC adsorbent is stronger than the 8%Fe_3_O_4_@MAC adsorbent. In the low-concentration Cr(VI) ion solution, there were many highly active sites on the surface of the adsorbent, which were more than the amount of initial Cr(VI) ions in the solution, so the collision between Cr(VI) ions and active sites increased, and the adsorption capacity of the adsorbent increased distinctly. However, in the different situation for the high-concentration Cr(VI) ion solution, the amount of active sites on the surface was not adequate, so it was difficult to adsorb so many Cr(VI) ions on the surface of adsorbent; thus, the curve tended to stabilize.

### 3.3. Effect of Fe_3_O_4_ Loading

The adsorption performances by different adsorbents are listed in Table 5, and the advantage of Fe_3_O_4_@MMC is more distinct than that of Fe_3_O_4_@MAC. The adsorption capacity of adsorbents reached 132.80 mg·g^−1^, and the adsorption rate of Cr(VI) ions was as high as 99.60% when the Fe_3_O_4_ loading was 8%. Moreover, the Fe_3_O_4_ loading mass percentage of every adsorbent by ICP-OES characterization results is close to the planned setting. The specific gravity of magnetic particles can affect the adsorption performance; 6% loading was insufficient, and 10% or 15% was excessive and inappropriate for the adsorbent. Too many magnetic particles might block the micropores, as was consistent with the BET results for the characterization of the adsorbent. The data were analyzed from the atomic absorption spectrometry were closer to the results; the error was very small, and the gap could be ignored.

### 3.4. Adsorption Isotherm Analysis

**Figure 10:**
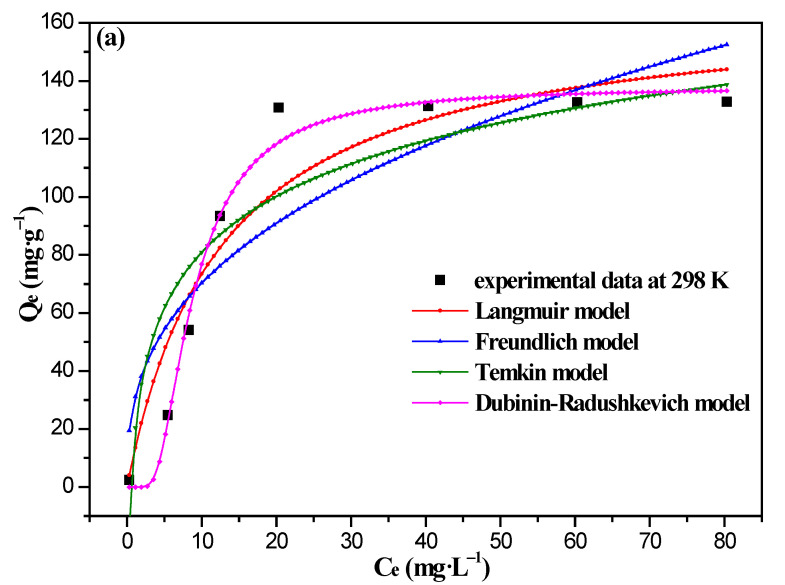

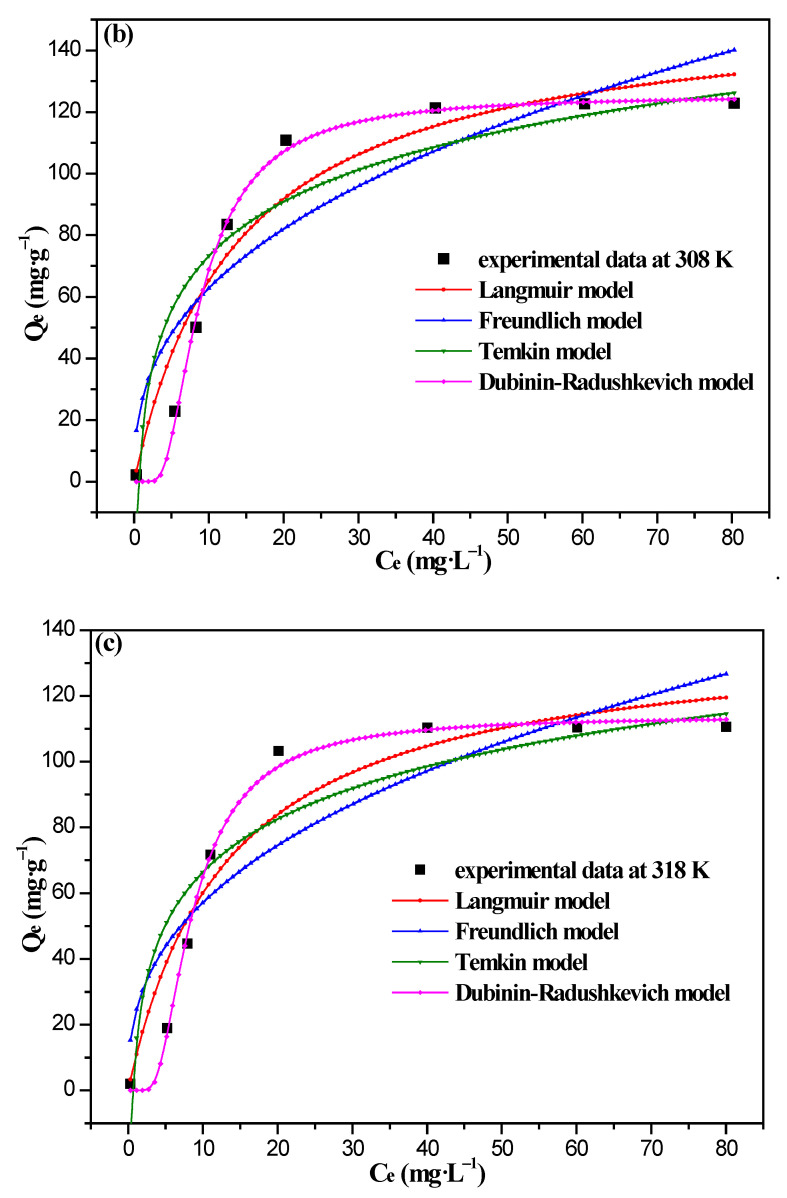
The fittng curves with nonlinear two-parameter adsorption isotherm models: (**a**) 298 K, (**b**) 308 K, and (**c**) 318 K.

The four kinds of adsorption isotherm models at different temperatures are chosen to analyze the real adsorption process on the 8%Fe_3_O_4_@MMC adsorbent in Figure 10. The fitting curves’ shapes are different, as is which are consisted with the different adsorption regular model.

Four kinds of adsorption isotherm models can fit the experimental data of the 8%Fe_3_O_4_@MMC adsorbent at 298 K, 308 K, and 318 K. However, the Dubinin–Radushkevich model were described better, so the adsorption tended to occur on the surface layer of the adsorbent, the adsorbed adsorbate had not interacted with the adsorbate, and the surface of the adsorbent was not uniform. The fitting parameters and correlation coefficients are listed in Table 6.

At the different temperature, the correlation coefficients (R^2^) of the Dubinin–Radushkevich model was closer to 1.0, and the value of Q_D_ was closer to the true experimental value; so, the surface of adsorbent is inhomogeneous, and the value of A_D_ exceeds 40 kJ·mol^−1^, thus indicating that this adsorption behavior belongs to chemisorption. The adsorption isotherm analysis of the 8%Fe_3_O_4_@MAC adsorbent was similar to the activated carbon, and the typical physical adsorption were reported widely in the literature [4,12,15,16,48].

Additionally, the separation factor (R_L_) can be used to distinguish whether the adsorption is favorable or not in the Langmuir model. We also calculated the value of R_L_ at different temperatures by the following equation [51]:

$$R_{L}=\frac{1}{1+C_{0}K_{L}}$$

The values of the R_L_ were 0.1366, 0.1462, and 0.1412 at 298 K, 308 K, and 318 K, respectively. All of the values of R_L_ are less than 1.0, indicating that this adsorption is favorable and 8%Fe_3_O_4_@MMC was an effective adsorbent for Cr(VI) ions’ adsorption.

### 3.5. Effect of Adsorption Time and Adsorption Kinetics

**Figure 11:**
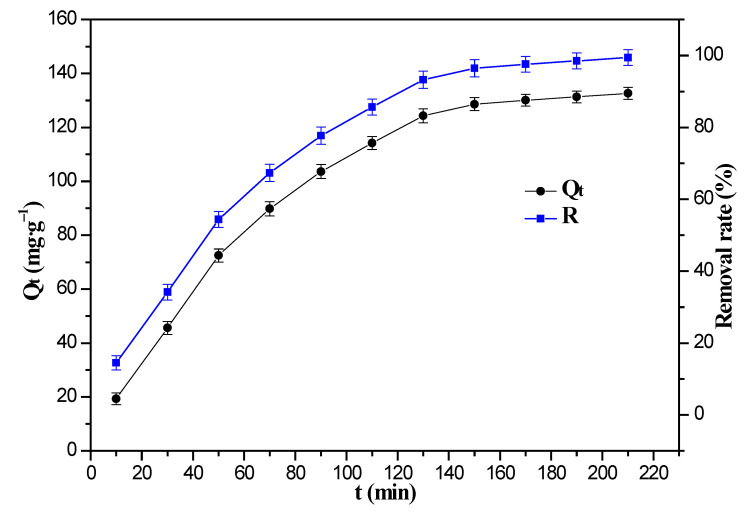
Effect of adsorption time on the uptake of Cr(VI) ions. Adsorption conditions: the initial Cr(VI) ions’ concentration, 80 mg·L^−1^; volume, 50 mL; pH, 2.0; adsorbent dosage, 0.03 g; temperature, 298 K.

The effect of adsorption time on the uptake of Cr(VI) ions by the 8%Fe_3_O_4_@MMC adsorbent is shown in Figure 11. When the adsorption time is below 130 min, the adsorption capacity (Q_t_) and the removal rate (R) rapidly increase distinctly. However, when the adsorption time exceeds 130 min, the removal rate of Cr(VI) ions increases slowly, and this might be due to the reduction of adsorption active sites. The adsorption capacity value was almost unchanged since the adsorption time exceeded 180 min, so the adsorption reached the dynamic equilibrium.

**Figure 12:**
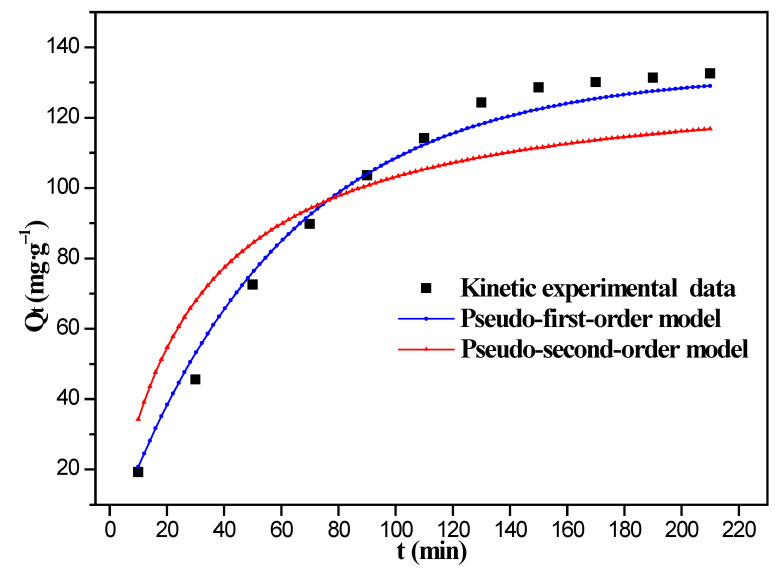
Fitting curves of adsorption data with two nonlinear kinetic equations by the 8%Fe_3_O_4_@MMC adsorbent. Adsorption conditions: the initial Cr(VI) ions’ concentration, 80 mg·L^−1^; volume, 50 mL; pH, 2.0; adsorbent dosage, 0.03 g; temperature, 298 K.

Adsorption kinetics models were investigated according to Equations (3) and (4), and fitting curves of adsorption data with two nonlinear kinetic equations by the 8%Fe_3_O_4_@MMC adsorbent are displayed in Figure 12. The corresponding fitting curves by the 8%Fe_3_O_4_@MAC adsorbent are shown in Supplementary Figure S5. The pseudo-first-order model is closer to the real kinetic experimental data at 298 K.

The parameters of adsorption kinetic models are listed in Supplementary Table S1 [50]. The correlation coefficient (*R*^2^) of the pseudo-first-order model is 0.9863, and the correlation coefficient (R^2^) of the pseudo-second-order model is 0.8472, so the pseudo-first-order model can better describe the real adsorption behavior. The value of *Q*_e (cal)_ is closer to the real experimental value in the pseudo-first-order model at 298 K. Hence, the physical adsorption and chemisorption might coexist, and the chemisorption was inapparent. Cr(VI) ions in solution free moving to the surface of adsorbent, and the stirring or vibration maybe is beneficial to this adsorption.

### 3.6. Thermodynamic Analysis

The thermodynamic analysis for Cr(VI) ions adsorption by the 8%Fe_3_O_4_@MMC and 8%Fe_3_O_4_@MAC adsorbent were investigated according to Equations (5) and (6) at different temperatures (from 288 to 328 K), and the results are listed in Table 7. It is clear that the Cr(VI) ions’ adsorption capacity of adsorbent decreases with the increment of temperature. The high temperature is not helpful for the adsorption of Cr(VI) ions, but the adsorption rate is low at a lower temperature, so 298 K is the optimal temperature. The hydrolysis of Cr(VI) ions occurred easily at a high temperature, so the low temperature is suitable to this adsorption by this adsorbent [52].

**Figure 13:**
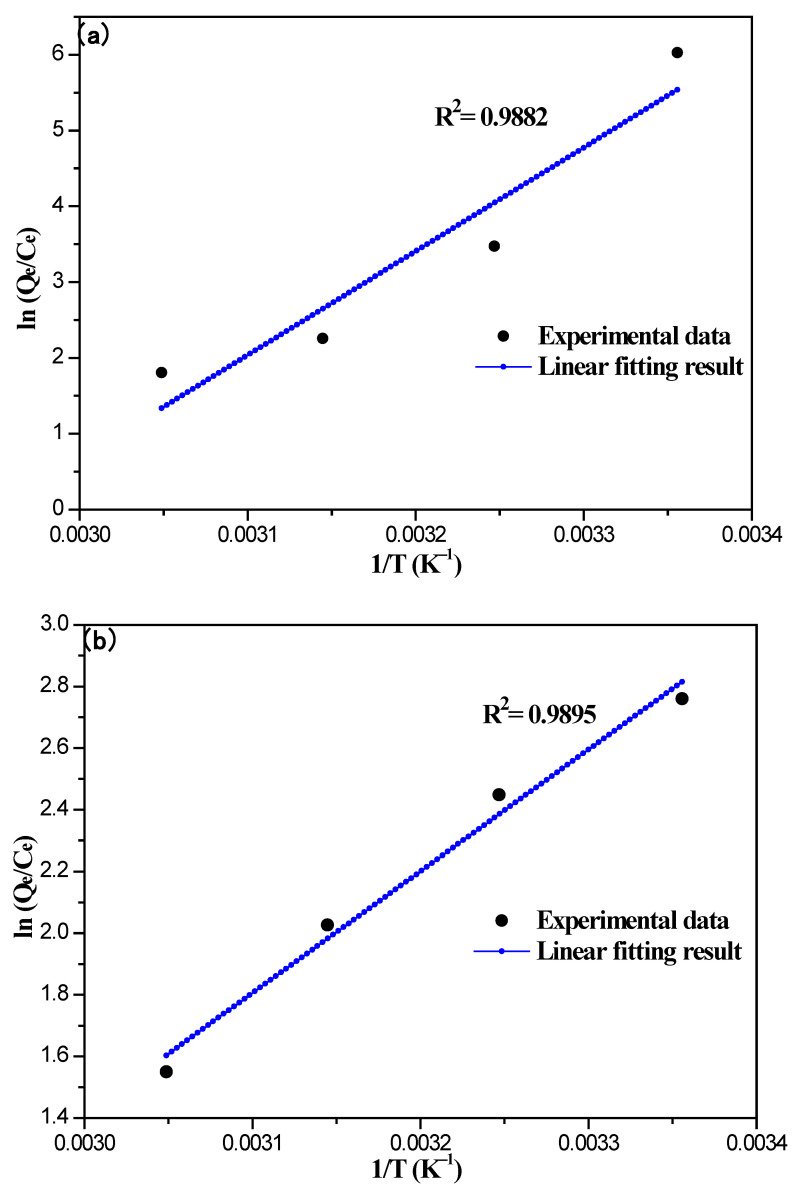
The plot dependence of ln(Q_e_/C_e_) on 1/T: (a) 8%Fe_3_O_4_@MMC and (b) 8%Fe_3_O_4_@MAC. Adsorption conditions: the initial Cr(VI) ions’ concentration, 80 mg·L^−1^; volume, 50 mL; pH, 2.0; adsorbent dosage, 0.03 g; adsorption time, 180 min.

The plot dependence of ln(Q_e_/C_e_) on 1/T is displayed in Figure 13, and the correlation coefficients are R^2^ = 0.9882 and R^2^ = 0.9895 according to the data analysis. The values of ΔH^0^ and ΔS^0^ of the 8%Fe_3_O_4_@MMC adsorbent are obtained from Equations (5) and (6) and are −113.832 kJ·mol^−1^ and −335.953 J·mol^−1^·K^−1^, and the value of ΔH^0^ and ΔS^0^ of the 8%Fe_3_O_4_@MAC adsorbent are obtained from Equations (5) and (6) are −32.827 kJ·mol^−1^ and −86.752 J·mol^−1^·K^−1^. The ΔH^0^ values indicate the adsorption process was exothermic in nature and may be governed by the enthalpy. The negative values of ΔS^0^ indicate that the adsorption of a cationic metal results in a decrease in overall entropy in the system. All the values of ΔG (Table 7) are negative at different temperatures, so the adsorption Cr(VI) ions process of these adsorbents is spontaneous, and a high temperature is not helpful for adsorption.

### 3.7. Reutilization of Adsorbent

**Figure 14:**
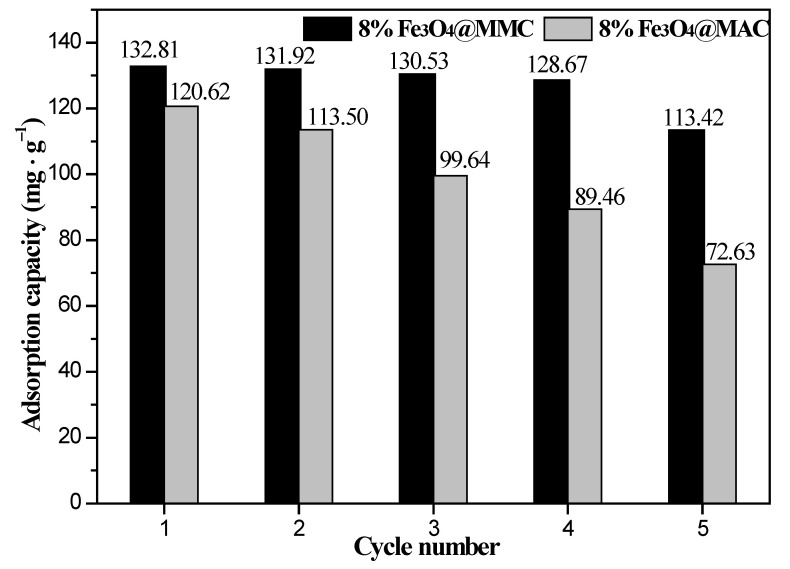
Repeated use of the 8%Fe_3_O_4_@MMC and 8%Fe_3_O_4_@MAC adsorbents. Adsorption conditions: the initial Cr(VI) ions’ concentration, 80 mg·L^−1^; volume, 50 mL; pH, 2.0; adsorbent dosage, 0.03 g; temperature, 298 K; adsorption time, 180 min.

In order to evaluate the economic value and application prospects, the recycling performance of the 8%Fe_3_O_4_@MMC and 8%Fe_3_O_4_@MAC adsorbent are displayed in Figure 14. After the adsorption of Cr(VI) ions, the adsorbent was transferred into the eluent solution (1 mol·L^−1^ H_2_SO_4_) for 6 h, and the desorption of the adsorbent was performed carefully. In the cyclic experiments, considering a little mass loss is inevitable, we chose to perform more repeated experiments to reduce the error, and the result in Figure 14 is the average value of five repeated experiments. The adsorption capacity of Cr(VI) ions by the 8%Fe_3_O_4_@MMC adsorbent was still as high as 113.42 mg·g^−1^, and the corresponding removal rate of Cr(VI) ions was still as high as 85.05% after five cycles. Hence, both the desorption capacity and reuse capacity of the 8%Fe_3_O_4_@MMC adsorbent are good.

The adsorption capacity of Cr(VI) ions by the 8%Fe_3_O_4_@MAC adsorbent decreased to 72.63 mg·g^−1^ after five cycles, and the corresponding removal rate of Cr(VI) ions was as low as 54.45% after five cycles. Actually, the adsorption capacity of Cr(VI) ions cut down fast to 99.64 mg·g^−1^, and the removal rate of Cr(VI) ions decreased to 74.71% with the third reuse. Thus, the recycling performance of this adsorbent was not ideal.

This strange phenomenon might be ascribed to the microstructure of the surface carbon material. Uniform pore structure might be helpful to develop the chemical bonding between carboxyl groups and Cr(VI) ions [30]. The irregular micropore structure of the surface carbon material was blocked easily, losing the good desorption and reuse capacity. Hence, the good desorption capacity and good reuse capacity of the 8%Fe_3_O_4_@MMC adsorbent was affirmed, and it showed great application prospects.

Many adsorbents containing magnetic carbon materials such as Fe-BDC@AC were prepared for the adsorption of Cr(VI) ions in water solution, and the maximum adsorption capacity for Cr(VI) ions in water solution by the typical adsorbents are listed in Table 8. The advantage of this adsorbent was distinct and affirmative due to the fact that the adsorption capacity for Cr(VI) ions reached 132.80 mg·g^−1^, and all of these experiments were performed by using a diluted sample from petrochemical wastewater. Hence, this adsorbent could be applied well for the removal of Cr(VI) ions in petrochemical wastewater.

### 3.8. Possible Adsorption Mechanism

The results showed that Dubinin–Radushkevich model isotherm matches well with the experimental data. The adsorption kinetic study reveals that the adsorption of Cr(VI) ions to 8%Fe_3_O_4_@MMC adsorbent obeys the pseudo-first-order equation. These results suggest that the uptake of Cr(VI) is most likely due to physical adsorption and chemisorption coexisting. Therefore, the surface area is one important characteristic, and surface properties must play a larger role in the physical adsorption process.

Electrostatic interaction between Cr(VI) ions and magnetic particles are strong due to the negative charge of Cr(VI) ions and the positive surface charge of adsorbents (containing Fe(III)). The electrostatic attraction could be helpful for Cr(VI) ions were adsorbed on the surface of this adsorbent at acidic solution. A similar trend is reported on Cr(VI) ions on Fe-BDC@AC [59].

The active functional groups on the surface of this adsorbent can also play an important role, part of the C=C reacted with Cr(VI), so part of Cr(VI) is reduced to Cr(III). Finally, Cr(III) is present in the form of Cr(OH)_3_, and this result is similar to the research in the literature [61]. At the same time, the reactive groups (C-C,C-O) in the carbon layer are also oxidized easily by Cr(VI), and all of the highly toxic Cr(VI) is transformed into Cr(III). This viewpoint is consistent with the carbonaceous adsorbents acting as electron donors to reduce Cr(VI) to Cr(III) [62]. So, the carbon layer of magnetic carbon material is the main reason for Cr(VI)’s adsorption performance.

Coordination bonds were formed between the oxygen element and Cr(VI) ion, due to the 8%Fe_3_O_4_@MMC adsorbent, included abundant oxygen elements from carboxyl (-COOH). However, the coordination capacity of Cr(VI) ions was limited due to the steric effect of oxygen atoms in Cr_2_O_7_^2−^, HCrO_4_^−^, or CrO_4_^2−^ ions [63]. However, the weak coordination bond also can be formed between Cr(VI) and many carboxyls on the surface of magnetic carbon materials. So, chemisorption exists in this Cr(VI) adsorption process.

In all, the construction of affluent useful active sites and porous structures in the carbon layer contributes to the adsorption of Cr(VI) in petrochemical wastewater. Compared to the 8%Fe_3_O_4_@MAC adsorbent, the advantage of the 8%Fe_3_O_4_@MMC adsorbent was obvious. The uniform pore structure was favorable for the adsorption stability in the petrochemical wastewater solution, and which averted the block in the adsorption process [33,64]. As with the nano magnetic carbon materials, 8%Fe_3_O_4_@MMC exhibited a greater adsorption performance and good reuse capacity, which had far surpassed 8%Fe_3_O_4_@MAC. So, the Fe_3_O_4_@MMC adsorbent can be applied for the industrial treatment of Cr(VI) ions in the petrochemical wastewater.

## 4. Conclusions

A series of the Fe_3_O_4_@MMC and Fe_3_O_4_@MAC adsorbents were prepared and applied for the removal of Cr(VI) ions in petrochemical wastewater solution successfully.

The maximum adsorption capacity of the 8%Fe_3_O_4_@MMC adsorbent reached 132.80 mg·g^−1^ by the Chinese national standards GB/T 7467-87 method, and the corresponding removal rate of Cr(VI) ions was 99.60% under the initial Cr(VI) ions’ concentration of 80 mg·L^−1^, volume of 50 mL, pH of 2.0, adsorbent dosage of 0.03 g, temperature of 298 K, and adsorption time of 180 min. The characterization results showed that the reasonable magnetic homogeneous dispersed, unique pore size, good thermal stability, and good micromorphology of the 8%Fe_3_O_4_@MMC adsorbent.

The adsorption conditions were optimized, adsorption isotherm models, kinetic and thermodynamic studies were analyzed. The physical adsorption and chemisorption coexisted in this adsorption process, and the stirring or vibration was beneficial to this adsorption. The adsorption Cr(VI) ions process was spontaneous and exothermic. The good desorption capacity and good reuse capacity of this adsorbent were affirmed. The preparation process of this adsorbent is easy. An 8%Fe_3_O_4_@MMC adsorbent can exhibit a great application prospect for Cr(VI) ions’ cleanup in petrochemical wastewater solution.

## Figures and Tables

**Table 1:** Comparison of adsorption capacity for Cr(VI) ions by typical carbon material.

Adsorbents	Q _max_ (mg·g^−1^)	Optimum pH	Years	References
Activated carbon from tires	48.1	2.0	2001	[21]
Activated carbon	112.36	1.0	2007	[22]
Pomegranate husk carbon	35.2	1.0	2009	[23]
Carbon slurry	15.24	2.0	2010	[24]
Carbon nanotube	9.0	2.0	2011	[25]
Graphene nanocomposites	1.03	2.0	2012	[26]
Carbon fabrics	5.59	1.0	2014	[27]
Biochar	24.6	2.0	2015	[28]
Rubberwood sawdust activated carbon	44.05	13.9	2018	[29]

**Table 2:** Equations of the isotherm models used in this study.

Models	Equation	Parameters
Langmuir	Q_e_ = $\frac{Q_{L}K_{L}C_{e}}{1+K_{L}C_{e}}$	Q_L_ (mg·g^−1^) maximum adsorption capacity; K_L_ (L·mg^−1^) Langmuir constant.
Freundlich	Q_e_ = K_F_$C_{e}^{1∕n_{F}}$	K_F_ (mg·g^−1^)(L·mg^−1^)^−1/n^ Freundlich constant; 1/n_F_ heterogeneity factor.
Temkin	Q_e_ = A_T_ + B_T_lnC_e_	A_T_ Temkin constant;B_T_ adsorption energy factor.
Dubinin–Radushkevich	Q_e_ = $Q_{D}\exp\{-A_{D}{\left[\ln(1+\frac{1}{C_{e}})\right]}^{2}\}$	Q_D_ (mg·g^−1^) adsorption capacity; A_D_ Dubinin–Radushkevich free energy constant.

**Table 3:** Textural properties of some samples.

Absorbent	Surface Area (m^2^·g^−1^)	Average Pore size (nm)	Pore Volume (cm^3^·g^−1^)	Average Particle Size (nm)
MAC	1303.0	1.82	2.41	17.45
6%Fe_3_O_4_@MAC	1306.3	1.82	2.43	19.27
8%Fe_3_O_4_@MAC	1311.3	1.81	2.45	21.23
10%Fe_3_O_4_@MAC	1307.6	1.79	2.39	23.46
15%Fe_3_O_4_@MAC	1304.3	1.78	2.30	26.33
MMC	436.7	6.82	0.35	16.34
6%Fe_3_O_4_@MMC	441.6	6.81	0.36	17.83
8%Fe_3_O_4_@MMC	452.3	6.80	0.38	18.37
10%Fe_3_O_4_@MMC	450.7	6.67	0.31	19.42
15%Fe_3_O_4_@MMC	448.4	6.63	0.25	20.06

**Note:** Average diameter of particle size determined by the nitrogen adsorption–desorption.

**Table 4:** The SEM–EDS analysis results of 8%Fe_3_O_4_@MMC adsorbent after adsorption.

Element	Weight Percentage (%)	Atom Percentage (%)
C	41.10	53.01
O	45.04	43.69
Fe	11.31	3.12
Cr	2.55	0.18
Total	100	100

**Table 5:** The adsorption performance by different adsorbents.

Absorbents	Adsorption Capacity (mg·g^−1^)	Removal Rate of Cr(VI) Ions (%)	Adsorption Capacity ^a^ (mg·g^−1^)	Fe_3_O_4_ Loading ^b^ (wt.%)
6%Fe_3_O_4_@MMC	126.37	94.78	126.51	5.97
6%Fe_3_O_4_@MAC	116.15	87.11	116.33	5.96
8%Fe_3_O_4_@MMC	132.80	99.60	132.94	7.99
8%Fe_3_O_4_@MAC	120.61	90.46	120.72	7.98
10%Fe_3_O_4_@MMC	124.03	93.02	124.15	9.97
10%Fe_3_O_4_@MAC	114.76	86.07	115.01	9.96
15%Fe_3_O_4_@MMC	116.49	87.37	116.82	14.98
15%Fe_3_O_4_@MAC	108.54	81.41	109.05	14.96

Adsorption conditions: the initial Cr(VI) ions’ concentration, 80 mg·L^−1^; volume, 50 mL; pH, 2.0; adsorbent dosage, 0.03 g; temperature, 298 K; adsorption time, 180 min. ^a^ The data were analyzed from atomic absorption spectrometry method. ^b^ The data were calculated based on the ICP–OES results.

**Table 6:** Fitting parameters and correlation coefficients with nonlinear two-parameter adsorption isotherm models at different temperatures.

Temperature/K	298	308	318
Langmuir model	K_L_ = 0.079	K_L_ = 0.073	K_L_ = 0.076
	Q_L_ = 166.788	Q_L_ = 154.770	Q_L_ = 139.171
	R^2^ = 0.892	R^2^ = 0.923	R^2^ = 0.912
Freundlich model	K_F_ = 29.844	K_F_ = 29.296	K_F_ = 23.694
	n_F_ = 2.689	n_F_ = 2.596	n_F_ = 2.614
	R^2^ = 0.779	R^2^ = 0.821	R^2^ = 0.807
Temkin model	A_T_ = 17.109	A_T_ = 14.672	A_T_ = 13.200
	B_T_ = 27.723	B_T_ = 25.431	B_T_ = 23.132
	R^2^ = 0.802	R^2^ = 0.829	R^2^ = 0.820
Dubinin–Radushkevich Model	Q_D_ = 137.954	Q_D_ = 125.430	Q_D_ = 113.884
	A_D_ = 64.682	A_D_ = 66.275	A_D_ = 61.638
	R^2^ = 0.988	R^2^ = 0.997	R^2^ = 0.996

**Table 7:** Effect of temperature and the Gibbs free energy change at different temperature.

Temperature /K	8%Fe_3_O_4_@MMC			8%Fe_3_O_4_@MAC		
	Adsorption Capacity (mg·g^−1^)	Removal Rate (%)	ΔG (kJ·mol^−1^)	Adsorption Capacity (mg·g^−1^)	Removal Rate (%)	ΔG (kJ·mol^−1^)
288	128.93	96.70	−9.31	118.33	88.75	−6.17
298	132.80	99.60	−14.94	120.61	90.46	−6.84
308	126.77	95.08	−8.89	116.54	87.41	−6.27
318	113.52	85.14	−5.97	109.32	81.99	−5.36
328	104.68	78.51	−4.93	98.49	73.87	−4.23

Adsorption conditions: the initial Cr(VI) ions’ concentration, 80 mg·L^−1^; volume, 50 mL; pH, 2.0; adsorbent dosage, 0.03 g; adsorption time, 180 min.

**Table 8:** Comparison of adsorption capacity for Cr(VI) ions in water solution.

Adsorbents	Carbon Form	Q _max_ (mg·g^−1^)	Years	References
GAC-QPVP	granular activated carbon	53.7	2007	[53]
MN	mesoporous magnetic carbon	3.74	2014	[54]
ZVI/carbon	magnetic carbon	275.6	2015	[55]
β-FeOOH/SYBK	activated carbon	37.04	2017	[56]
Fe_3_O_4_/C	synthesize magnetite carbon from potassium fulvic acid	64.0	2019	[57]
AC/SPION	activated carbon	16.82	2019	[58]
Fe-BDC@AC	activated carbon	100	2022	[59]
AgNPs/GO/Chitosan	graphene	46.45	2022	[60]
8%Fe_3_O_4_@MMC	mesoporous magnetic carbon	132.80	in this work	

## Data Availability

Data are contained within the article or Supplementary Materials. The data presented in this study are available in the Supplementary Materials.

## Supplementary Materials

The following supporting information can be downloaded at https://www.mdpi.com/article/10.3390/nano12213815/s1. Figure S1: Exhibits N_2_ adsorption–desorption isotherms of the 8%Fe_3_O_4_@MMC adsorbent. Figure S2: Displays pore size distributions of the 8%Fe_3_O_4_@MMC adsorbent. Figure S3: Exhibits N_2_ adsorption–desorption isotherms of the 8%Fe_3_O_4_@MAC adsorbent. Figure S4: Displays pore size distributions of the 8%Fe_3_O_4_@MAC adsorbent. Figure S5: Exhibits the fitting curves of adsorption data with two nonlinear kinetic equations by the 8%Fe_3_O_4_@MAC adsorbent. Table S1: Shows the parameters of adsorption kinetic and intraparticle diffusion model.
