# Supplementary material for: Investigation of the Adsorption Process of Chromium (VI) Ions from Petrochemical Wastewater Using Nanomagnetic Carbon Materials
Source: Nanomaterials (Basel). 2022 Oct 28;12(21):3815. doi: 10.3390/nano12213815 (PMC9653853; doi:10.3390/nano12213815)
Supplement: Supplementary file 1 [file nanomaterials-12-03815-s001.zip › nanomaterials-1919715-supplementary.pdf]

Supplementary Materials

Investigation of the Adsorption Process of Chromium (VI) Ions from Petrochemical Wastewater using Nanomagnetic Carbon Materials

Wei Long [1,2,*], Zhilong Chen [2], Xiwen Chen [2] and Zhanye Zhong [2]

[1] Guangdong Provincial Key Laboratory of Petrochemical Pollution Process and Control, Guangdong University of Petrochemical Technology, Maoming 525000, China
[2] College of chemistry, Guangdong University of Petrochemical Technology, Maoming 525000, China
[*] Correspondence: longwei@gdupt.edu.cn

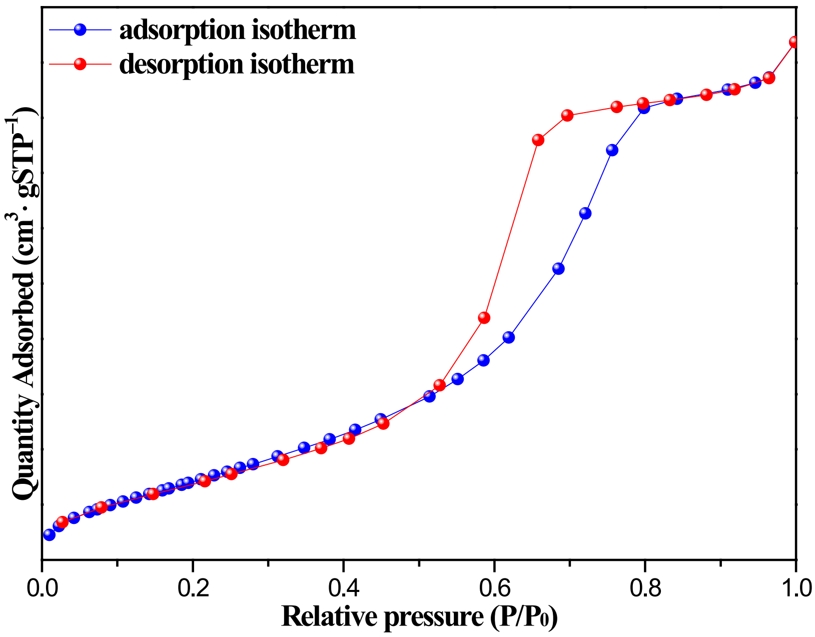

Figure S1. N_2 adsorption-desorption isotherms of the 8%Fe_3O_4@MMC adsorbent.

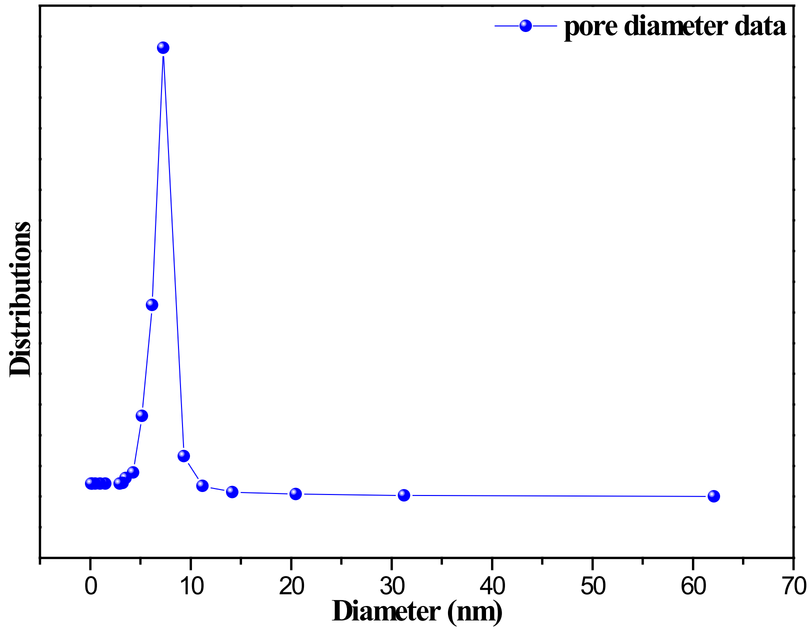

Figure S2. Pore size distributions of the 8%Fe_3O_4@MMC adsorbent.

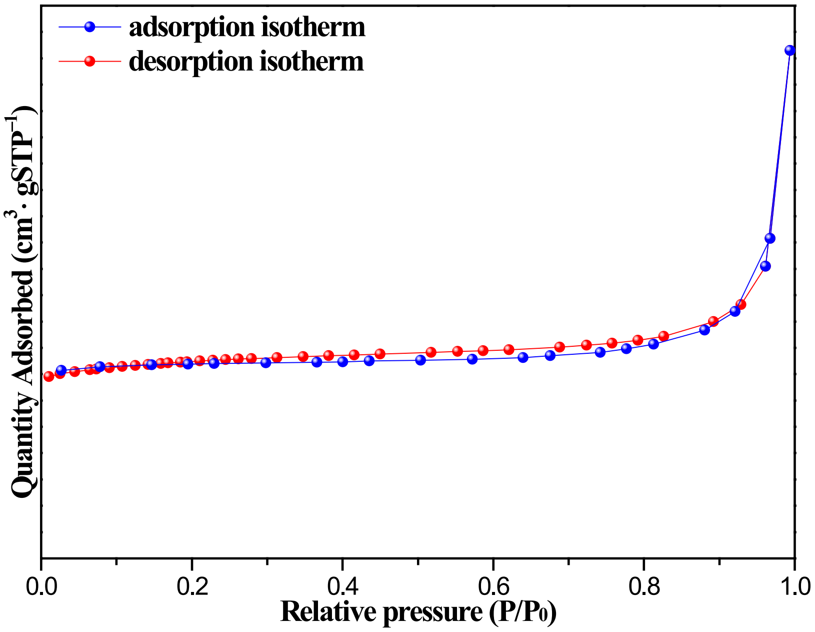

Figure S3. N_2 adsorption-desorption isotherms of the 8%Fe_3O_4@MAC adsorbent.

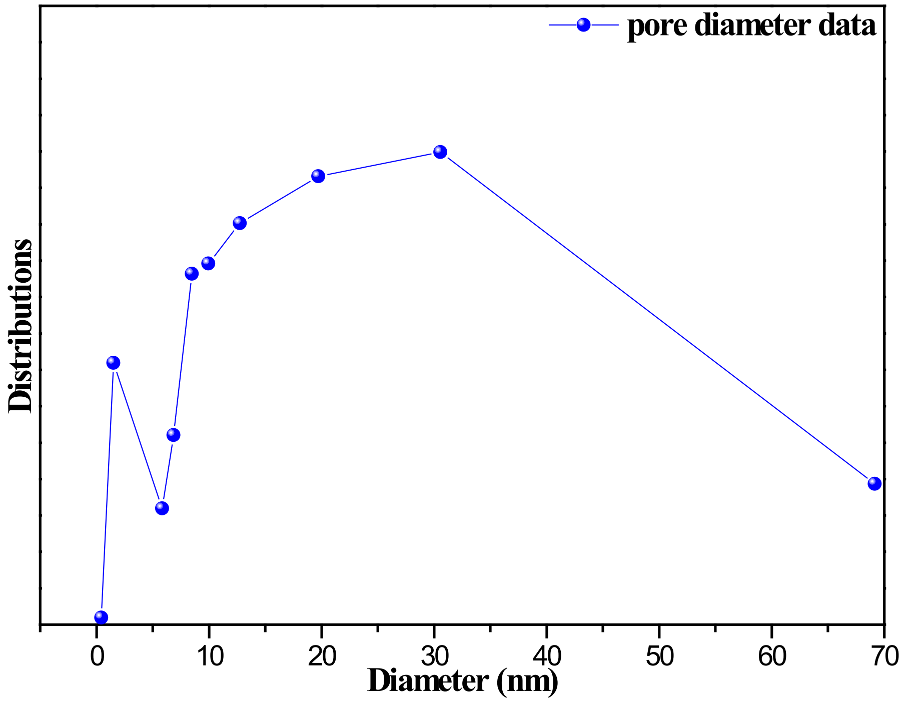

Figure S4. Pore size distributions of the 8%Fe_3O_4@MAC adsorbent.

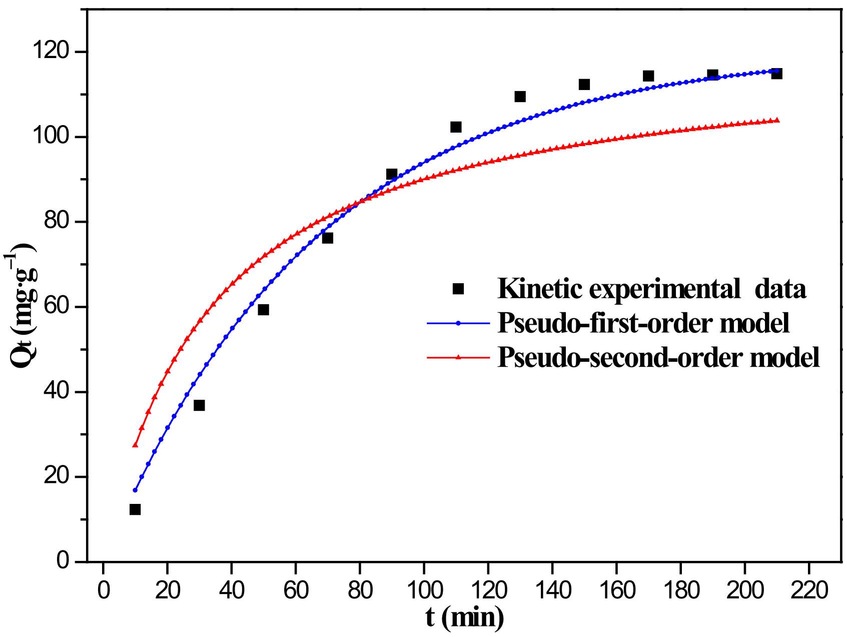

Figure S5. Fitting curves of adsorption data with two nonlinear kinetic equations by 8%Fe_3O_4@MAC adsorbent. Adsorption conditions: the initial Cr(VI) ions concentration, 80 mg·L^{-1}; volume, 50 mL; pH, 2.0; adsorbent dosage, 0.03 g; temperature, 298 K.

Table S1. Parameters of adsorption kinetic models.

Models	Parameters	8%Fe_3O_4@MMC	8%Fe_3O_4@MAC
Pseudo-first-order equation	$Q_{e\,(cal)}$ (mg/g)	131.56	120.43
	k_1 (min^{-1})	0.0170	0.0151
	R^2	0.9863	0.9855
Pseudo-second-order equation	$Q_{e(cal)}$ (mg/g)	141.35	124.14
	k_2 (g·mg^{-1}·min^{-1})	2.62×10^{-4}	2.44×10^{-4}
	R^2	0.8472	0.8606

$Q_{e\,(cal)}$ is calculated by the linear fitting analysis.
